# The Monkeypox Pandemic as a Worldwide Emergence: Much Ado?

**DOI:** 10.3390/idr14040064

**Published:** 2022-08-10

**Authors:** Massimo Ciccozzi, Nicola Petrosillo

**Affiliations:** 1 Unit of Medical Statistics and Molecular Epidemiology, University Campus Bio-Medico, 00128 Rome, Italy; 2 Infection Prevention and Control, University Hospital Campus Bio-Medico, 00128 Rome, Italy

The dramatic emergence of SARS-CoV-2 infection worldwide opened the Pandora’s box of fears and frights for new emerging infections. When the first human cases of monkeypox were reported in Europe in travellers from Africa in May 2022, and later on also in close contacts of them with no history of travels abroad, the fear of a new epidemic became more evident.

Monkeypox is a zoonotic infection caused by monkeypox virus (MPXV) [1,2], which is endemic in several African countries [3,4]; indeed, it has been primarily detected in Western and Central Africa. Although the name of this disease suggests that monkeys are the primary host, the specific animal reservoir of MPXV remains unknown [5]. Some evidences suggest that native African rodents such as Gambian giant rats *(Cricetomys gambianus)* might be a natural reservoir of the virus [6,7].

From an epidemiological point of view, MPXV was first isolated in 1958 from skin lesions during an outbreak of vesicular disease among captive cynomolgus macaques imported from Singapore into Denmark for polio-vaccine-related research [5].

In 1970, the first human isolate of MPXV was reported in a child in the equatorial region of the Democratic Republic of the Congo (DRC), nine months after the eradication of smallpox in that country [3]. The first outbreak of MPX reported outside of Africa [8] was linked to importation of infected mammals in 2003 into the United States. Since 2018, only 12 travel-associated MPXV cases were reported outside Africa until the end of 2021. In May 2022, for the first time, many outbreaks were reported worldwide (in non-endemic countries) without any epidemiological link to travel or imported mammals [9].

As of 25 July 2022, 9697 confirmed cases of human monkeypox infection have been reported from 27 EU/EEA countries, mostly in Spain (3151), Germany (2352), France (1567), the Netherlands (712), Portugal (588), and Italy (407) [10].

The incubation period for MPX is usually 6 to 13 days but can range from 5 to 21 days [9]. This disease often begins with a combination of the following symptoms: fever, headache, chills, exhaustion, asthenia, lymph node swelling, back pain, and muscle aches [11]. Commonly, within three days after onset of symptoms, maculopapular rash starts from the site of primary infection and rapidly spreads to other parts of the body. Palms and soles are involved in cases of the disseminated rash. The lesions progress, usually within 12 days, often asynchronously from the stage of macules to papules, vesicles, and pustules, before falling off [12].

In the current outbreaks, the clinical manifestations in travel-related cases detected in Western countries have usually been mild, and many cases presented with rashes in the anogenital region [13].

MPX is mostly a self-limited disease, typically lasting two to four weeks and more often with complete recovery [8]. MPX virus transmission occurs through contact with an infected animal or human or contact with material contaminated with the virus [14].

Before 2022, virus transmission through direct or indirect contact with live or dead animals was assumed to be the main factor for human MPX infections. This may occur by bite or scratch, direct contact with body fluids, or lesions from an infected animal or contaminated material (indirect contact) [14].

Human-to-human transmission of MPXV was considered to occur mostly through respiratory droplets during close and prolonged face-to-face contact, by direct contact with body fluids of an infected person, or contact with contaminated objects [2,14]. In a reporting MPX outbreak in Nigeria in the 2017, sexual transmission was hypothesized as a plausible route of infection, as it involved close skin-to-skin contact during sexual inter-course or transmission via genital secretions [15].

In 2022, with the vast majority of cases in men identifying as men who have sex with men (MSM) and with histories pointing to exposure during sexual intercourse, transmission through sexual contact was found to be the main driver of these outbreaks.

In a few weeks, the disease—long a concern in some African countries—has spread worldwide; recently, the World Health Organization has declared monkeypox a global health emergency.

Should we be worried? In this outbreak, the reproduction number (R0) for MPXV seems to be higher than 1; therefore, in high-risk populations, including MSM, an expanding outbreak is likely [16]. On the other side, more than 16,000 people have been infected worldwide and 5 persons have died (i.e., case fatality rate 0.03%), overwhelmingly belonging to the MSM community. Therefore, the low case fatality rate, the relatively limited number of infections outside African countries where MPX is endemic, and the self-limiting clinical course of infection could lead to the spread of MPXV only being faced with careful contact tracing, monitoring, information, and education of high-risk groups. Should it work for MPXV infections? What is the lesson learned by other sexually transmitted diseases, including HIV?

The next step is vaccination. In the new WHO interim guidance on vaccine and immunization against MPX, the WHO proposes vaccination as post-exposure and pre-exposure prophylaxis in contact of cases within 4 days from the exposure and in clinical laboratory staff working on MPX and in other at-risk populations, respectively [17].

In addition, do we have a vaccine against MPXV? People vaccinated against smallpox before 1980 should have some immunity against monkeypox. In 1988, Fine et al. estimated an 85% cross-protective action of the small pox vaccine against monkeypox [18]. However, over the years, immunity waves.

The WHO stated that a smallpox vaccine should not be used widely for tackling this MPX outbreak because there is limited clinical data, and above all, there is a limited supply of these vaccines (WHO). However, there is a vaccine against smallpox (Imvanex) that has been licensed by the FDA to be used for Monkeypox; moreover, EMA (European Medicine Agency) recommends approval of Imvanex for the prevention of MPX in high-risk groups.

The evolution of the MPX outbreak is worrisome, principally because the epidemic does not seem to slow down; however, we should be aware that this is not the dreadful epidemic that will replace COVID-19, but something should still be done soon. Less Ado and More Interventions.
